# Rapid mimicry of trunk and head movements during play in African Savanna elephants (*Loxodonta africana*)

**DOI:** 10.1038/s41598-025-01067-2

**Published:** 2025-05-09

**Authors:** Giada Cordoni, Martin Hecker, Valentina Crippa, Beatriz Gallego Aldama, Santiago Borragán Santos, Ivan Norscia

**Affiliations:** 1 Department of Life Sciences and Systems Biology, University of Torino, Turin, Italy; 2 Cantur, S.A. Parque de la Naturaleza de Cabárceno, 39690 Obregón (Cantabria), Spain

**Keywords:** Rapid motor mimicry, Play contagion, Competitive play, Emotional contagion, *Loxodonta africana*, Animal behaviour, Social evolution, Emotion

## Abstract

The basic forms of motor and possibly emotion replication include behavioral contagion and rapid motor mimicry (RMM). RMM—mainly demonstrated during play—occurs when an individual perceives and rapidly (< 1 s) replicates the exact motor sequence of another individual. We collected data on an African Savanna Elephant (*Loxodonta africana*; *N* = 15) group housed at the Parque de la Naturaleza de Cabárceno (Spain) on play target movements of both trunk and head. We demonstrated the presence of RMM. Elephants that were more prone in mimicking others’ target movements were also more prone to play after observing others playing. RMM—as behavioral contagion—can enhance action coordination between players. As RMM was associated with more offensive play patterns than unreplicated target movements, RMM may allow competitive play sessions to occur, possibly replacing agonistic interactions. Neither individual (age, sex) nor social (affiliation levels) factors modulated the RMM. These findings can be related to the elephant high tolerance levels and the wide presence of play across age (including adults) and sex. Concluding, African elephants have the potential to share their affective states (emotional contagion) via RMM which is relevant to the investigation of the evolution of empathy in mammals including humans.

**Supplementary Information:**

The online version contains supplementary material available at 10.1038/s41598-025-01067-2.

## Introduction

The ability to replicate the actions or movements of others relies on embodied mirroring mechanisms, where observing others’ actions can influence one’s own actions, and performing one’s own actions can modulate the perception of others’ actions^1^. Such ability requires that the representational resources subserving the production of own action also subserve the perception of foreign action^1,2^. Mirroring others’ actions is important as shared circuits for actions can be coupled with shared circuits for perceiving and experiencing the emotions conveyed by the actions^3^. Observed and perceived emotional actions convey information to the observer that allows him/her to replicate the observed action and the sensory and affective state underlying it^4^. The most basic forms of motor replication include automatic behavioral contagion and rapid motor mimicry, possibly mediated by the mirror neuron system^4–7^. Behavioral contagion and rapid motor mimicry both occur when an individual observes the motor patterns of another individual and automatically replicates them^1,6,8^. In the case of rapid motor mimicry, the exact motor sequence is mirrored, and the replication occurs within 1s (500 ms in humans^5,6,9^).

In non-human mammals, rapid motor mimicry has been studied mostly in relation to the relaxed open mouth display or play face during playful interactions (for review:^9^; but see^10^ for the mimicry of bared teeth display). In particular, rapid facial mimicry has been found in dogs^11^, meerkats^12^, sun bears^13^ and various primate species spanning humans^14^, great apes (bonobos:^10^; chimpanzees:^15^; lowland gorillas:^15,16^; Bornean orangutans:^17^), and monkeys such as geladas^18,19^ and Tonkean and Japanese macaques^20^.

According to the Perception-Action Model, motor mimicry can vary depending on the observer’s experience (e.g. associations for a specific kind of target, situation, state) which produces variation across individuals^4^. Indeed, individual factors (such as sex and age) and social factors (such as social bond and kinship) can modulate rapid motor mimicry, as it has been seen in relation to rapid facial mimicry (see^9^). For example, juvenile/adolescent orangutans and playmates 2–7 years apart were found to respond to their playmates’ facial displays with the same facial expression^17^. In bonobos, the occurrence of rapid motor mimicry of silent bared-teeth display was affected by the sex of the partners with female homosexual contacts being punctuated by a higher presence of rapid motor mimicry^10^. In humans, individuals tend to mimic the facial expressions of in-group members more than those of out-group members^21^. Also in dogs, it was observed that the higher the level of affiliation, the higher the rate of rapid motor mimicry^11^.

Regarding the possible function of rapid motor mimicry with respect to playful interactions, it has been found that rapid motor mimicry can be linked to longer play sessions. For example, in dogs, humans, apes and monkeys the presence of rapid motor mimicry can be associated with longer durations of playful sessions, indicative of higher success of play interactions (e.g.,^11,12,15,17,22,23^). Rapid motor mimicry can be associated with the more or less competitive nature of the play session, allowing for the management of more vigorous sessions, especially in tolerant species, where play can be used for non-aggressive competition (e.g., spider monkeys;^23^), or for rebalancing the session, especially in less tolerant species (e.g., lowland gorillas;^16^).

Elephants are long-lived and large-brained mammals that live in complex and tolerant social environments, where they must keep track of their relationships with many partners^24,25^. They display complex and multimodal communication, cooperation, comforting behaviors, and social learning from conspecifics^24,26–29^. Many of these features are shared with primates, including humans^30^. Therefore, investigating in elephants the occurrence of behavioral phenomena underlying empathy, such as rapid motor mimicry, may contribute valuable insights to the broader puzzle of the evolution of empathic behavior in social mammals, including humans. Elephants are particularly suitable to investigate rapid motor mimicry in the context of play also because they are a playful species, where social play is maintained in adulthood^26,31^. Furthermore, in the group of African Savanna elephants (*Loxodonta africana*) observed in this study it has been demonstrated that elephants would start to play most often after observing other elephants playing (i.e. play contagion^32^;).

Most literature on rapid mimicry in non-human animals has focused on the relaxed open mouth display or play face, specific play signals (for a review see^9^). However, in elephants the open mouth display may not be the most suitable pattern to investigate mimicry. This display is associated with vocalization, and it is also shown during agonistic encounters (not just play), when an elephant can hold their mouth open while chasing an opponent^27,33,35^. Moreover, the trunk results from the fusion of the nose and the upper lip, and sensory and motor components of the trunk also control the rest of the face (nerve branches innervating both trunk and upper/lower lips;^36–39^). As a result of this anatomical dependency, the open mouth is associated with trunk raising^36,40^. Thus, we focused on play specific trunk and head movements to investigate rapid motor mimicry in African elephants.

### Prediction 1—presence of rapid motor mimicry

Motor mimicry falls within the motor replication phenomena, as behavioral contagion, and it has been particularly observed in relation to play signals (e.g.^12,15,17,18,23^). In the study group, social play contagion was found by a previous study^32^. Therefore, we expected to find also rapid motor mimicry of trunk/head movements during play in the group under study.

### Prediction 2—factors modulating rapid motor mimicry

Play is pervasive in African elephants^26,31,41^ and play contagion was not influenced by individual factors, such as age and sex of the involved individuals^32^. Therefore, we expected no influence of sex and age of the players on the level of rapid motor mimicry (*Prediction 2a*). Rapid motor mimicry can be enhanced by social closeness (e.g.^11,12,42^). In the study group, social play contagion mainly occurred between individuals that affiliated the most^32^. Hence, we expected that also rapid motor mimicry would be more frequent in highly affiliating dyads than in low affiliating dyads (*Prediction 2b*).

### Prediction 3—play session features in presence of rapid motor mimicry

In various species, the presence of rapid motor mimicry (e.g. play face) during play has been associated with longer play sessions (e.g.^11,12,15,23^). Therefore, we expected that also in African Savanna elephants the presence of rapid motor mimicry can be linked to longer play sessions (*Prediction 3a*). Moreover, it has been observed in non-human primates that rapid motor mimicry may signal non-aggressive intent when play becomes more competitive and therefore it may be associated with more vigorous playful interactions^23^. Because in elephants play can be used in a non-aggressive competitive way as a result of the selection against aggression^41^, we expected that the occurrence of rapid motor mimicry may be associated with rougher playful patterns compared to the presence of non-replicated trunk/head movements only (*Prediction 3b*).

### Prediction 4—relation between rapid motor mimicry and play contagion

Both rapid motor mimicry and behavioral contagion are automatic, embodied mirroring phenomena possibly based on perception-action model^4^. In both cases, shared neural representations between the performer of the behavior and the observer allow the observer to replicate the behavior and socio-emotionally connect with the performer^3,4,7^. Hence, we expected that the individuals that were more prone to start playing after observing other individuals playing (i.e. play contagion) would be also more prone to rapidly mimic others’ play movements (trunk and head play signals).

## Results

### Preliminary result

Hourly frequencies of affiliation statistically differed between the different trigger-responder dyads (one sample chi-square *N* = 116, χ^2^ = 100.000, df = 15, *p* < 0.001; minimum, maximum, and mean ± SE values of dyadic affiliation levels: 0.0; 1.07; 0.068 ± 0.01).

### Prediction 1—presence of rapid motor mimicry

**Fig. 1:**
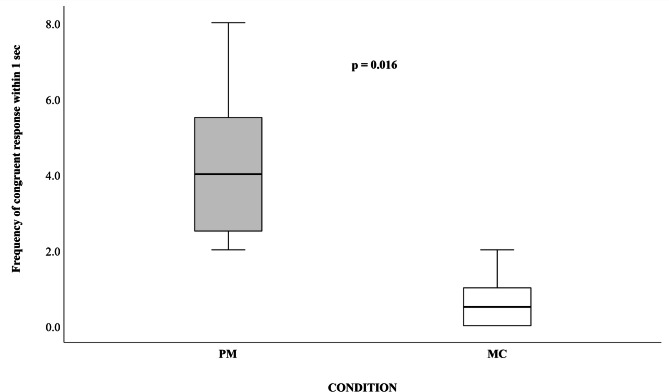
Boxplot showing in the Y-axis the frequency of congruent response (i.e. same triggering play target movement replicated within 1s) by the responder in the Post-Movement (PM; after the beginning of the triggering play target movement; light grey box) and in Matched-Control (MC; in absence of any previous triggering play target movement; white box) condition (X-axis).

It was significantly more likely that a potential responder replicated the same target movement of the trigger within 1s (congruent response) in Post-Movement (PM) than in Matched-Control (MC) condition (Wilcoxon Exact Test: ${\text{N}}_{{{\text{individuals\_exposed\_to\_target\_movements}}}}$N_individuals_exposed_to_target_movements_= 8, T = 0, ties = 1, *p* = 0.016; Fig. 1 and Video S1). Instead, not significant difference was observed between PM and MC in case of non-congruent response (when the potential responder showed one of the target movements but not the same movement as the trigger; Wilcoxon Exact Test: ${\text{N}}_{{{\text{individuals\_exposed\_to\_target\_movements}}}}$ = 8, T = 0, ties = 3, *p* = 0.063).

### Prediction 2—factors modulating rapid motor mimicry

GLMM_1_ was carried out on the individual (sex and age of trigger and potential responder), social factors (affiliation levels), and play session duration. The full model (including all fixed factors) and the null model (only including the random factor) did not significantly differ (likelihood ratio test: N_PM_dyads_ = 58, χ^2^ = 6.859, df = 6, *p* = 0.334). Hence, none of the tested variables explained the occurrence of RMM.

We ran a control model (GLMM_2_) on the data collected in MC condition. Also in this case, the full model did not significantly differ from the null model (N_MC_dyads_ = 58, χ^2^ = 11.813, df = 6, *p* = 0.07).

### Prediction 3—play session features in presence of rapid motor mimicry

**Fig. 2:**
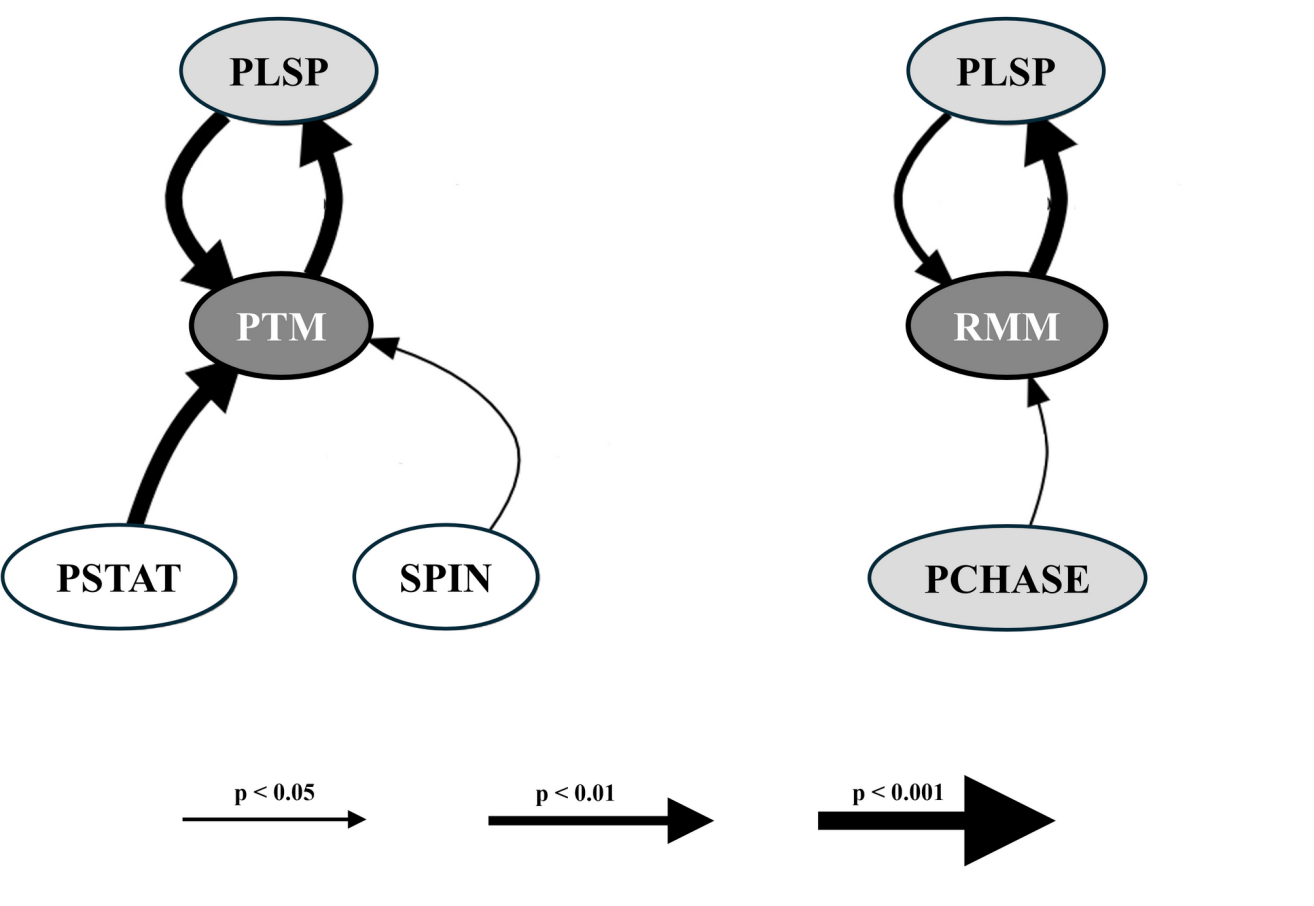
Flow diagram (Graphviz script) showing behavioral transitions across Rapid Movement Mimicry (RMM) and single Play Target Movement (PTM) not mimicked. In the white circles are represented neutral play patterns (PSTAT = play standing tall; SPIN = spinning) and in the light grey circles offensive play patterns (PLSP = play sparring; PCHASE = play chase). The thickness of the arrows indicates the level of significance of each transition.

A total of 48 and 108 transitions were recorded before and after Rapid Motor Mimicry (RMM) and single play target movements not mimicked, respectively. We found that RMM was most likely preceded and followed by play sparring and preceded by play chase (Fig. 2). Both patterns across RMM are offensive patterns. Instead, we found that play target movements not mimicked were most likely preceded and followed by play sparring (offensive pattern) and preceded by play standing tall and spinning, which are neutral patterns (Fig. 2).

### Prediction 4—relation between rapid motor mimicry and play contagion

**Fig. 3:**
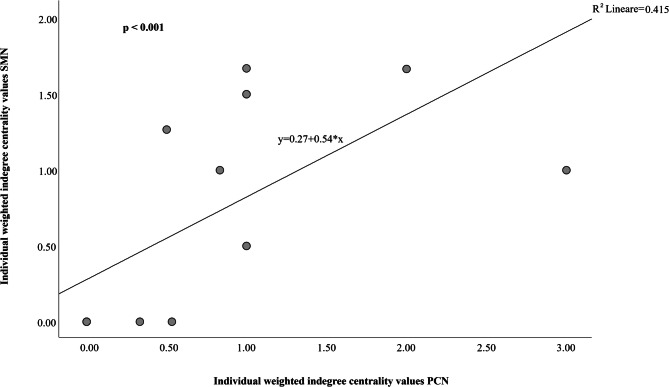
Scatterplot graph representing the positive correlation between individual weighted indegree centrality values of Social Mimicry Network (SMN; Y-axis) and individual weighted indegree centrality values of Play Contagion Network (PCN; X-axis).

The weighted indegree centrality values of elephants in the Social Mimicry Network (SMN) positively correlated with their weighted indegree centrality values in the social Play Contagion Network (PCN; Spearman’s correlation: ${\text{N}}_{{{\text{individuals}}}}$ = 15, *r* = 0.810, *p* < 0.001; Fig. 3). Hence, the individuals that were most central in showing RMM (by replicating the play target movements of companions) were also most prominent in being ‘infected’ by others’ play behavior (as they were most likely to start playing after that others had started play).

## Discussion

In the current study, we demonstrated for the first time the presence of rapid motor mimicry (RMM; *Prediction 1 confirmed*; see Video S1) in an Elephantidae species, namely savannah African elephants (*Loxodonta africana*). As a matter of fact, it was more likely that an elephant (i.e. the responder) performed a play target movement within 1 s after that a trigger had performed it, compared to the absence of any previous target movement performance. Furthermore, the responder was more likely to perform the exact target movement expressed by the trigger (congruent response) rather than another type of target movement (incongruent response). The presence of RMM is in line with previous findings showing that African elephants can ‘copy’ the play behavior of others which is another form of motor replication^6,32^. Indeed, behavioral contagion and RMM share similar neurobiological mechanisms probably including the mirror neuron system and the perception action model^4–7^. Consistently, the individuals that were most central in replicating the play target movements of others were also more prone to start playing after that others had started play (play contagion; *Prediction 4 confirmed*).

It has been previously shown that RMM (specifically, rapid facial mimicry) can enhance action coordination between players and the regulation of reciprocal play movements (e.g.^10,16,23^). African Savanna elephants are known to actively coordinate their movements with one another and communication exchange (e.g. rumble exchange;^43–45^). Because elephants can show complex playful interactions, RMM may be particularly useful to them to coordinate actions and manage the play session. Although we detected no association between the occurrence of RMM and play session duration (found instead in other species,^10–12,15,23^; *Prediction 3a not confirmed*), we found that RMM of the target movement was associated with more offensive patterns than the unreplicated target movements (*Prediction 3b confirmed*). This finding may explain why *Prediction 3a* was not confirmed, by reinforcing the hypothesis that RMM may be more useful in managing particularly competitive play sessions rather than in prolonging playful interactions. Although the sequential relation between RMM and offensive play patterns has been largely neglected, one study investigating this aspect found a similar result in spider monkeys (*Ateles* spp.;^23^). Spider monkeys share with elephants a fission-fusion, multi-male, multi-female society, the ability to engage in complex play sessions, and the involvement of adult individuals in playful interactions^25,26,44–46^. These elements suggest that RMM in elephants may function in allowing competitive play sessions to occur, possibly replacing agonistic interactions. Indeed, in the same group of African Savanna elephants a previous study found that social play may be used as a form of non-aggressive competition as a possible result of the selection against aggression^41^.

Neither individual (sex, age; *Prediction 2a confirmed*) nor social (affiliation levels; *Prediction 2b not confirmed*) factors affected the likelihood of observing RMM. The lack of an effect of age and sex is in line with previous findings of play contagion which is not affected by such factors^32^. This finding may arise from the fact that on one hand, the frequency of social play peaks during the juvenile phase^25,26,41^ but, on the other hand, the occurrence of behavioral contagion and rapid mimicry tend to increase with age in specific groups (e.g.,^47,48^). This increase may be associated with the development of neural networks responsible for interpreting social cues and understanding the internal states of others^47,48^, which might be essential for detecting the playful states of others. Furthermore, regarding sex social play is pivotal for both males and females: males typically use play to assess abilities of potential competitors, whereas females use play to build social bonds with kin^26^. Hence, both sexes probably have interest in coordinating their actions during play via RMM to make interaction more effective.

In the elephants under study, play contagion—another automatic phenomenon possibly underlying emotional contagion^4^—was positively influenced by the level of affiliation between the individual who initiated the first play session (trigger) and the individual who was not initially involved in play but started a new session (responder) after observing the trigger playing^32^. No such familiarity bias was found for RMM in the same population, in this study. An important difference between play contagion and RMM is that contagion does not occur with the individual already involved in the play session, but rather with another individual who is not yet engaged. Behavioral contagion therefore operates beyond the dyad that is currently interacting and may serve to synchronize and coordinate with others^49^, and particularly socially proximate individuals^9^. RMM, on the other hand, occurs within the play session, acting as a *hic-et-nunc* phenomenon between the two players. It may help manage a challenging exchange of patterns and prevent misunderstandings during the interaction(^23^; present study). Further studies on a larger dataset are necessary to determine whether the lack of a familiarity effect on RMM in African savanna elephants is generalizable or not. Another explanation may be that the elephants in our study probably had a high degree of familiarity with one another, even if they belonged to different families, and this could potentially mask the effect of affiliation levels on RMM. However, the dyadic affiliation levels varied significantly between dyads (see “Preliminary result”). This variation in affiliation levels may allow testing for a possible influence on the occurrence of RMM. Further investigation and an expanded database also included wild African elephants may reveal possible effects of individual and social factors (including kinship) on RMM that have not emerged in this study.

In conclusion, even though our study was carried out on a relatively small group of captive African Savanna elephants, it demonstrated for the first time the occurrence of RMM in this species. Hence, elephants might have the potential to share their affective states via RMM, which might provide the ground for future investigation of possible convergences and/or homologies with human emotional contagion.

## Methods

### The study group

**Fig. 4:**
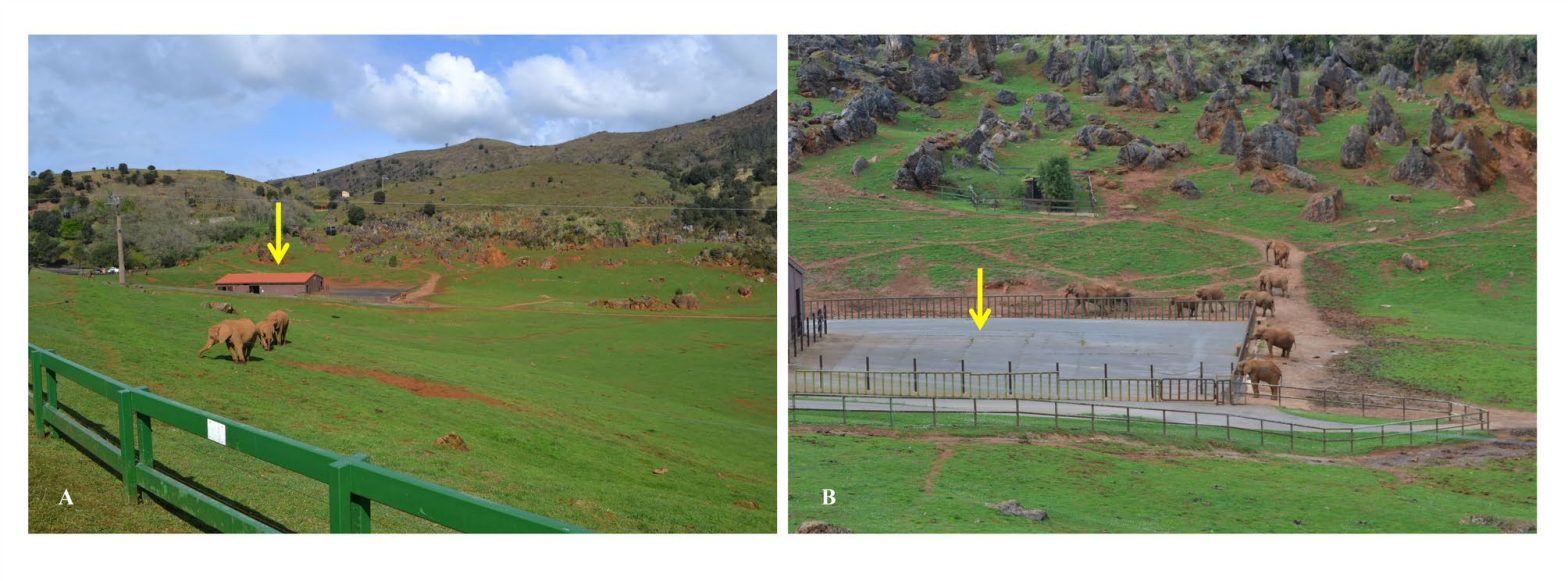
Pictures showing the elephant natural habitat outdoor space of 25 ha at the Parque de la Naturaleza de Cabárceno (Spain). Night cages are indicated with a yellow arrow.

This research was conducted on a colony of 15 African Savanna elephants (*Loxodonta africana*) housed at the Parque de la Naturaleza de Cabárceno (Santander, Cantabria, Spain), in a natural habitat outdoor space of 25 ha (see Fig. 4). The colony was composed of six immature subjects (four elephants of 2–5 years old and two elephants of 10–11 years old at pre-pubertal stage), two late adolescents (17–19 years old), and seven adults (21–45 years old; age classes as for^47^; see Table 1 for colony composition). Three out of the 15 elephants (Jums, Penny, and Zambi) were born in the wild, and only one adult male (Jumar) was born outside of Cabárceno (see Table 1). The first elephant to be housed at Cabárceno was Penny, in 1990. The individuals composing the group during our study had been kept together for nearly 10 years. Elephants would remain outdoors the whole day and would spend the night indoors. Several elevated viewpoints permitted the observation of most or all elephants outdoor.

**Table 1:** The composition of the African savanna elephant group under study.

Individual	Sex	Age	Father (in the colony)	Mother (in the colony)	Offspring
Jums	M	45			
Penny	F	42			
Zambi	F	41			Kenia, Infinita
Kira	F	27		Penny	Africa (Pamba)
Kenia	F	21		Zambi	
Infinita	F	17		Zambi	Toranzo
Brisa	F	19			Toribio
Hilda	F	21			Martin, Saja, Maruca
Africa (Pamba)	F	11		Kira	
Toranzo	M	4	Jumar	Infinita	
Toribio	M	4	Jumar	Brisa	
Saja	F	5	Jumar	Hilda	
Maruca	F	2	Jumar	Hilda	
Martin	M	10	Jums	Hilda	
Jumar	M	25	Jums		

### Data collection

Behavioral data were collected outdoors on a daily basis (4–6 days/week) from April to July 2022, from 9:00 am to 6:00 pm (with morning/afternoon shifts). Observations were carried out live by M.H. with the support of full HD audio-videos (via Panasonic HC-V180). Elephants were individually recognized based on sex, size, and physical features (e.g. ear cuts, tail length). Audio-video data on affiliation and social play sessions between elephants were collected via all occurrences sampling methods^50^ on the visible individuals. We extracted 30 h of videos on dyadic social play (number of analyzed play sessions: 188) out of 91 h of video footage. The ethogram used for this study is summarized in Table 2. Play sessions were analyzed frame-by-frame or in slow-motion by using Avidemux 2.8.1. Behavioral coding was carried out by M.H. and V.C. after training with I.N. and G.C., and the inter-observer reliability measured via Cohen’s k was 0.80 (good agreement;^51^).

**Table 2:** The social play ethogram of the African savanna elephants under study (from^32,41^).

Pattern	Description
Social play contact patterns:	This item includes the following: (i) **american football** (offensive pattern): elephants try to catch an object by play fighting (e.g. pushing aside, pulling) with one or more fellows; (ii) **clamber** (offensive pattern): elephants clamber onto others forming a pile of wriggling, squirming elephants; (iii) **kneel-down** (neutral pattern): an elephant lower himself down on his knees and playfully spars with a smaller partner; (iv) **play mock charge** (offensive pattern): an elephant withdraws and then runs towards the fellow and pushes it; (v) **play push** (offensive pattern): an elephant exerts force on a fellow with a part of its body; (vi) **play retrieve** (offensive pattern): an elephant pulls back another with its trunk; (vii) **play slap** (offensive pattern): an elephant hits another with its trunk; (viii) **play sparring** (offensive pattern): two elephants engage into head-to-head contact; (ix) **play trunk wrestle** (offensive pattern): two elephants entwine their trunks and push each other back and forth; (x) **tickle** (neutral pattern): an elephant stimulates another via repeated gentle contacts with its trunk (in association with at least another play pattern); (xi) **play fighting** (offensive pattern): two elephants engage in wrestle with no observable aggressive patterns
Social play non-contact patterns:	This item includes the following: (i) **play chase** (offensive pattern): an elephant rapidly follows another; (ii) **play flee** (defensive pattern): an elephant rapidly moves away from the partner which is chasing it; (iii) **play standing-tall** (neutral pattern): an elephant stands with the head held high while looking down over the tusks at an adversary; (iv) **play stretching head** (neutral pattern): an elephant stretches the head down and forward while gazing at a play partner; (v) **play tail raising** (defensive pattern): an elephant lifts its tail into air; (vi) **play water** (neutral pattern): various behaviors, such as swimming, splashing, skimming, submerging, head lifting performed in both solitary and social manner; (vii) **spinning** (neutral pattern): an elephant rapidly turns around
Play target movement:	This item includes the following: (i) **circus pose**: an elephant lifts and holds its trunk up in an S-shape, similar to play trunk periscope, but with upper bend of the trunk resting against the elephant’s forehead; (ii) **flop trunk on head**: an elephant puts its floppy trunk on its own head from a raised position; (iii) **head waggling**: an elephant moves its head from side to side; (iv) **play forward trunk swing**: an elephant swings or tosses its trunk toward an adversary, (v) **play trunk periscope**: an elephant pauses and approaches a group mate with the trunk held up in a periscope or S-shape position

### Operational definitions

*Play session*. A social play session was characterized by the behavioral patterns reported in Table 2 (from^41^). A play session started when one individual initiated a playful pattern with a companion and ended when either player disengaged from each other (for at least 30s) or an uninvolved elephant interrupted the interaction^41^.

**Fig. 5:**
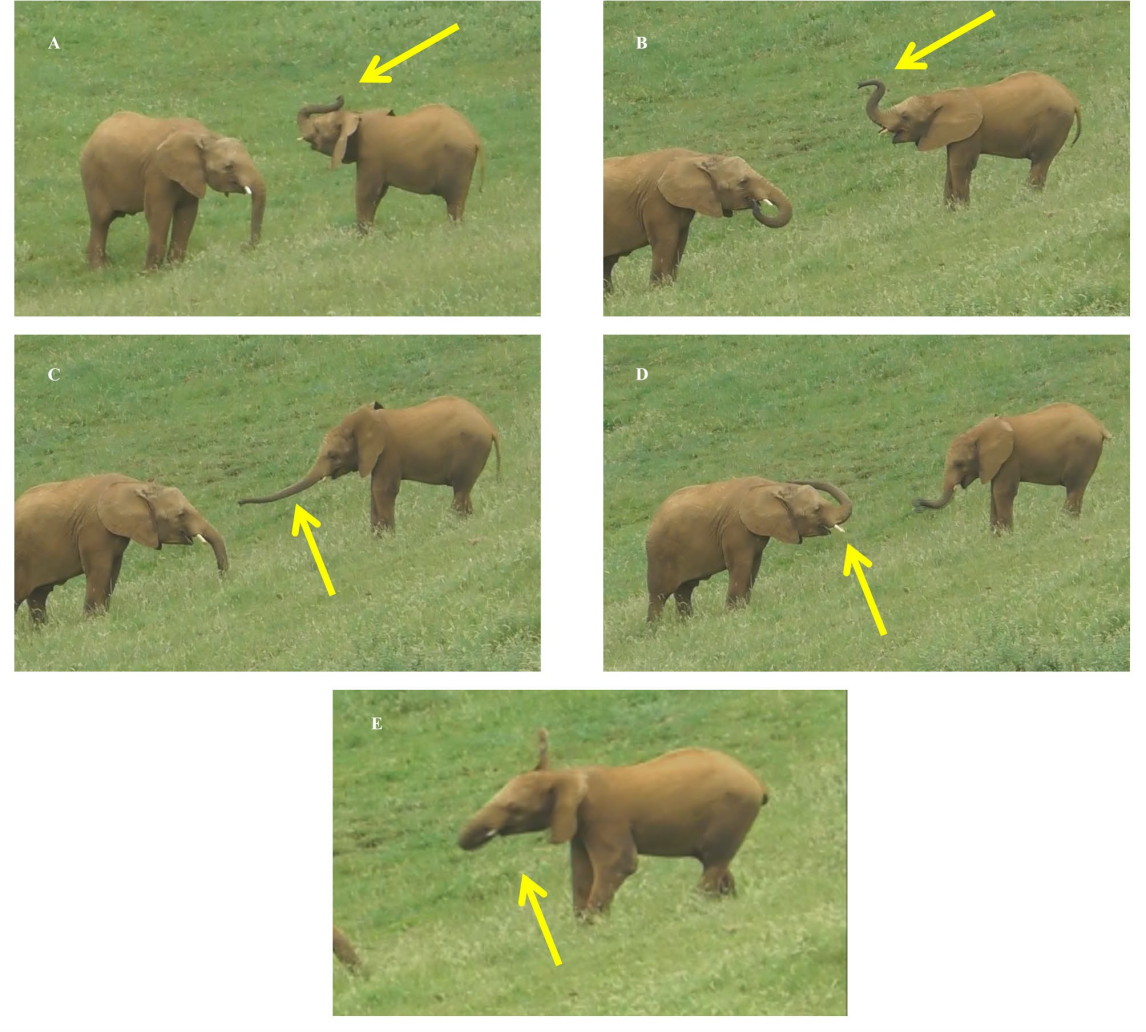
Pictures showing the play target movements (indicated with yellow arrows) considered in this study: circus pose (A), play trunk periscope (B), play forward trunk swing (C), flop trunk on head (D), and head waggling (E). Each play target movement is described in detail in the Table 2.

*Play target movements*. Within each play session we considered the following movements to check for the presence of mimicry: Play Forward Trunk Swing (PFTS), Play Trunk Periscope (PTP), Circus Pose (CP), Flop Trunk on Head (FTH), and Head Waggling (HW) (see Table 2 for definition; Fig. 5 for the different movements). These movements (hereafter, play target movements), four of which involving the trunk, are considered as play markers^26,27,35^. We considered the start of the play target movement as the first video frame in which the individual moved the trunk or head from a neutral position (head still or trunk lowered), and the end of the target movement as the last video frame in which the head and trunk returned to the neutral position.

*Rapid Motor Mimicry evaluation method*. During a dyadic playful interaction, the first elephant performing a play target movement was labeled as ‘trigger’ and the other playmate was labeled as ‘potential responder’. Potential responder could replicate the same motor pattern performed by the trigger (congruent response), perform one of the other play target movements but not the same of the trigger (incongruent response) or not perform any of the target movements (no response). The potential responder could see the triggering play session and was not involved in any other social interactions. To check for the presence of Rapid Motor Mimicry (RMM; response latency < 1s) of the target movements, we applied a modified version of the Post-Conflict/Matched Control (PC-MC) method, originally defined to check for post‐conflict reunions in animals^52^ and recently applied to check for grooming and yawn contagion^48,52,53^. In particular, we identified two conditions: (1) Post-Movement (PM) and Matched Control (MC). In the PM condition the trigger performed one of the play target movements. After the beginning of the triggering movement, we checked the potential responder to record whether they would replicate the triggering movement (thus showing a response movement) within 1s. In the MC condition, within the same session (therefore in the same exact conditions as the PM), at a time point t_0_, we checked whether in absence of a previous triggering play target movement the same potential responder as PM showed a response movement within 1s. To ensure motor pattern independence and reduce sequence bias between PM and MC, t_0_ of MC was selected at a time distance of at least 10s before or after the PM.

### Social networks

Via freeware Gephi 0.9.7 (www.https://gephi.org/, distributed under the dual license CDDL 1.0 and GNU General Public License v3;^54^), we determined the Social Mimicry Network (SMN) and the social Play Contagion Network (PCN), both including the network actors (nodes) and the relations between them (edges). Social play contagion in the study group was demonstrated by Norscia and colleagues^32^ and it occurred when an individual (responder) started playing after a previous play session had been started by other individuals (triggers) in the previous three minutes. The social networks were obtained from the number of cases of RMM per dyad (SMN, directed edges: A→B if A was the play initiator and B the recipient; B→A if the other way around) and dyadic events of play contagion (PCN, directed edges: A→B if A was the trigger and B the responder; B→A if the other way around) and normalized over the number of opportunities to show mimicry and observe contagion for that dyad, respectively.

Via Social Network Analysis, we calculated the SMN and PCN weighted indegree centrality values. Degree centrality results from the number of neighbors of each node; specifically, in-degree centrality values derive from the number of direct connections received by other nodes. Thus, the most central node is that receiving the most direct connections^55^. Weighted centrality considers, for each node, the weight of ties among nodes^56^.

### Statistical analyses

Owing to the small sample size (*N* < 10), we applied the Exact Wilcoxon signed-rank test to compare the individual frequency of either congruent response (when the potential responder replicated the same play target movement of the trigger) or non-congruent response (when the potential responder showed one of play target movements but not the same movement as the trigger) between PM and MC^57^. In these analyses, we included only the individuals (*N* = 8) that were exposed to at least one of the target movements during play.

We applied a one sample chi-square test to assess possible difference in hourly frequencies of affiliation between the different trigger-responder dyads.

On the data collected in PM condition (${\text{N}}_{{{\text{PM\_dyads}}}}$ = 58), we ran a Generalized Linear Mixed Model (GLMM_1_) to verify the possible association between individual factors, affiliation levels, and play session duration on the occurrence of RMM. We also ran a control model (GLMM_2_) on the data collected in MC condition (${\text{N}}_{{{\text{MC\_dyads}}}}$ = 58). In both models, we defined the occurrence of a play target movement as the target variable (binomial variable, presence = 1, absence = 0). We included the following fixed factors: i) (trigger’ and potential responder’s sex (binomial variable, male = 0, female = 1) and age (scale variable, years); (ii) dyadic affiliation levels (scale variable, hourly frequencies), and (iii) session duration (scale variable, seconds). We did not include relatedness between individuals (i.e. mother-offspring, siblings) as a fixed factor because the frequency of play between kin was very low. Furthermore, in the majority of cases play sessions between kin did not last long enough to select MC at a time point at least 10 s before or after PM.

Trigger and responder identities were included as random factors. Dyadic behavioral frequencies of affiliation were obtained by normalizing the affiliation bouts over the observation time (hours) of the interacting dyads. We fit the GLMMs in R via the function “glmer” of the R-package lme4^58–60^. We compared the full model including all fixed factors with the null model only including the random factors^60^. We used a likelihood ratio test^61^ to test the comparison significance (ANOVA with argument ‘Chisq’). We calculated the p values for the individual predictors based on likelihood ratio tests between the full and the null model by using the R-function “drop1”^62^. As the target variables were binomial, a binomial error distribution was used. We obtained the variance inflation factor (VIF) for the GLMM numeric variables via the “vif” function in R. All VIF values were ≤ 1.00, thus indicating no collinearity^63,64^.

For the sequential analysis, we generated a string for each RMM incident by recording the behavioral patterns and separating them with a break symbol (|). Similarly, for a control analysis, we generated the same type of string for each play target movement not mimicked (without RMM). This string represented the ordered sequence of behavioral patterns preceding and following either RMM or single play target movement not mimicked. Via the free, open-source software Behatrix 0.9.11 (http://www.boris.unito.it/pages/behatrix;^65^), we analyzed the behavioral sequences and organized data into contingency tables. The program then generated codes for a flow diagram (Graphviz script) showing behavioral transitions across RMM or single play target movement not mimicked. For the purpose of this analysis, we distinguished playful behavioral patterns as offensive (i.e. behavioral patterns aimed at attacking and pursuing the partner), defensive (i.e. behavioral patterns aimed at evading an attack, freeing oneself from a playmate contact or fleeing from the partner), and neutral (i.e. behavioral patterns neither offensive nor defensive;^23^; Table 2).

Finally, conducted a Spearman’s bivariate correlation (non-normal data distribution; 0.001 ≤ *p* ≤ 0.013) for the weighted indegree centrality values between SMN and PCN, obtained via social network analysis.

For all tests, the significant probability threshold was fixed at 0.05.

## Electronic supplementary material

Below is the link to the electronic supplementary material.

Supplementary Material 1

Supplementary Material 2

Supplementary Material 3

Supplementary Material 4

Supplementary Material 5

Supplementary Material 6

## Data Availability

The data sets supporting this article have been uploaded as part of the Supporting Material.
