# Supplementary material for: Rapid mimicry of trunk and head movements during play in African Savanna elephants (Loxodonta africana)
Source: Sci Rep. 2025 May 9;15:16263. doi: 10.1038/s41598-025-01067-2 (PMC12064687; doi:10.1038/s41598-025-01067-2)
Supplement: Supplementary file 6 — Supplementary Material 6 [file 41598_2025_1067_MOESM6_ESM.docx]

**Video S1** - An example of a Rapid Motor Mimicry event during contact play. The white arrow indicates the two players and the moment in which the responder rapidly (< 1sec) replicates the exact trunk play target movement (specifically, a play trunk periscope) of the trigger.
